# Connections of Grasping and Horizontal Hand Movements with Articulation in Czech Speakers

**DOI:** 10.3389/fpsyg.2017.00516

**Published:** 2017-04-05

**Authors:** Mikko Tiainen, Jiří Lukavský, Kaisa Tiippana, Martti Vainio, Juraj Šimko, Fatima Felisberti, Lari Vainio

**Affiliations:** ^1^Department of Psychology and Logopedics, University of HelsinkiHelsinki, Finland; ^2^Institute of Psychology, Czech Academy of SciencesPrague, Czechia; ^3^Department of Modern Languages, University of HelsinkiHelsinki, Finland; ^4^Kingston University LondonLondon, UK

**Keywords:** grasping, manual gestures, speech, manual actions, articulation

## Abstract

We have recently shown in Finnish speakers that articulation of certain vowels and consonants has a systematic influence on simultaneous grasp actions as well as on forward and backward hand movements. Here we studied whether these effects generalize to another language, namely Czech. We reasoned that if the results generalized to another language environment, it would suggest that the effects arise through other processes than language-dependent semantic associations. Rather, the effects would be likely to arise through language-independent interactions between processes that plan articulatory gestures and hand movements. Participants were presented with visual stimuli specifying articulations to be uttered (e.g., A or I), and they were required to produce a manual response concurrently with the articulation. In Experiment 1 they responded with a precision or a power grip, whereas in Experiment 2 they responded with a forward or a backward hand movement. The grip congruency effect was fully replicated: the consonant [k] and the vowel [α] were associated with power grip responses, while the consonant [t] and the vowel [i] were associated with precision grip responses. The forward/backward congruency effect was replicated with vowels [α], [o], which were associated with backward movement and with [i], which was associated with forward movement, but not with consonants [k] and [t]. These findings suggest that the congruency effects mostly reflect interaction between processes that plan articulatory gestures and hand movements with an exception that the forward/backward congruency effect might only work with vowel articulation.

## Introduction

Hand movements and mouth movements are connected. For example, when a participant is watching a large object being grasped while simultaneously pronouncing a syllable, the mouth is opened more than when watching a smaller object being grasped (Gentilucci, 2003). Additionally, when grasping an object while uttering an open vowel, the hand is opened wider than when a closed vowel is uttered (Gentilucci and Campione, 2011). The authors of these studies have proposed that these effects might reflect the functioning of neurons like the ones found in macaque monkeys that are active both when grasping an object with the hand or with the mouth (Rizzolatti et al., 1988). According to one version of the so-called gestural theory of language evolution, this interaction between mouth and hand actions may have originally operated for eating behavior, but gradually it might have provided neural ground to transferring communication from hand gestures to articulatory gestures (Gentilucci and Corballis, 2006). Indeed, some authors have even hypothesized that speech could have evolved from or alongside with communication based on manual gestures and grasping in particular (e.g., Rizzolatti and Arbib, 1998; Arbib, 2005).

In agreement with the above-mentioned gestural theories, the mouth-hand mimicry theories assume that people tend to mimic with mouth movements what their hands are doing (Paget, 1930; Hewes, 1973). It has been observed that young children (Forrester and Rodriguez, 2015) and chimpanzees (Waters and Fouts, 2002) tend to perform mouth movements, such as tongue protrusions, in imitative synchrony with fine-motor hand actions. Taking this view a step further, Ramachandran and Hubbard (2001) suggested that some articulations can even be thought of as mimes of hand actions. For example, words denoting smallness may involve narrowing of the vocal tract (e.g., “little” or “teeny”). According to these authors this movement could be seen as an analog of a manual precision grasp—which is used to pick up an object between the thumb and index finger—with a similarly narrow aperture. However, it has to be noticed that these views were not based on experimental evidence but on theoretical reasoning. Moreover, these connections do not, of course, apply to all words. Still, a recent analysis of over 4000 languages found that, for example, the vowel [i] is systematically associated with words denoting smallness (Blasi et al., 2016).

Based on the mouth-hand mimicry theories, we have hypothesized that planning certain articulatory gestures might be systematically integrated with planning precision and power grasp actions (Vainio et al., 2013). In general, grasping actions can be divided into precision and power grips (Napier, 1956) that have their own neural, functional and developmental properties (Halverson, 1931; Napier, 1956; Rizzolatti et al., 1987; Ehrsson et al., 2000). We developed a dual-action paradigm in which Finnish participants were required to pronounce a syllable (e.g., [ti]) and simultaneously respond either with a precision or a power grip response according to the color in which the syllable was written. We found that power grip responses are faster when they coincide with the pronunciation of the syllables [kα], [hα], or [ke] than with the syllables [ti], [hi], or [te]. When participants pronounce [ti], [hi], or [te], they respond with the precision grip more quickly than when they pronounce [kα], [hα], or [ke]. More recently we have shown that this grip congruency effect can be also observed in vocal responses so that the power grip response is associated with particularly rapid pronunciation of [kα] whereas the precision grip response is associated with rapid pronunciation of [ti] (Tiainen et al., 2017).

We have proposed that these associations demonstrate that the network for articulatory gestures partially overlaps with grasping networks (Vainio et al., 2014). In addition, we assume that they provide behavioral demonstration of some assumptions of the mouth-hand mimicry theories: certain articulatory gestures might be vocal counterparts of specific manual actions. We reasoned that the vowels [α] and [i] would be associated with the power and precision grip, respectively, because the mouth aperture is larger for [α] (an open vowel), similar to how the hand is opened wider when grasping something big with a power grip. In contrast, the mouth aperture is relatively small for [i] (a closed vowel), similar to the aperture between the thumb and index finger in the precision grip when grasping a small object.

When we originally selected the consonants [t] and [k] for the study (Vainio et al., 2013), we speculated that the voiceless alveolar stop [t] might be a suitable articulatory counterpart for the precision grip, since the tip of the tongue is utilized in [t] and the tips of the fingers are used in the precision grip. The voiceless velar stop consonant [k] was selected as an opposing pair for the consonant [t] because it is the only Finnish voiceless stop consonant that does not employ the tongue tip but instead is produced by raising the back of the tongue to contact with the soft palate. We assumed that the articulatory gesture for [k] might be viewed as an articulatory counterpart for the power grip action because the power grip is produced by moving the intermediate and proximal components of all fingers against the palmar surface of the hand, i.e., both actions use the base part of the effector.

In addition to the earlier mentioned analogs between a specific grip and articulatory gesture, Ramachandran and Hubbard (2001) also theoretically reasoned that words such as “you” might mimic pointing forward, as the articulation of that word requires pouting one’s lips forward. In contrast, articulatory gestures in some other words such as “I” might mimic pointing backward toward oneself. However, regardless of this potential interaction between articulatory gestures and corresponding horizontal hand movements, the research has focused on investigating how direction-related semantic content of a sentence or word interacts with horizontal hand movements. It has been, for example, shown that if participants have to judge whether a sentence makes sense, and the sentence implies action away from the body (e.g., “Close the drawer”), they move their hand away from their body faster. In contrast, hand movements toward the body are facilitated if the sentence implies action toward the body (e.g., “Open the drawer”) (Glenberg and Kaschak, 2002). Chieffi et al. (2009) have shown a similar effect with direction-related semantic information of a single word. They found that a hand movement pointing away from oneself is faster when a word meaning “there” (“LA”) is simultaneously read compared to when a word meaning “here” (“QUA”) is read. In contrast, hand movement pointing toward oneself is faster when a word meaning “here” is read compared to when a word meaning “there” is read.

We have recently demonstrated that forward and backward hand movements can be influenced by pronouncing a speech unit during the hand movement (Vainio et al., 2015). In this forward/backward congruency effect, participants move their hand faster forward when pronouncing [i] rather than [α] or [o]. Contrarily, they pull their hand back faster when pronouncing [α] or [o] rather than [i]. The effect also appeared in vocal responses; [i] was pronounced more quickly when paired with forward rather than backward movement, whereas [α] and [o] were pronounced faster when paired with a backward rather than forward movement. Similarly to the grip congruency effect, the forward/backward congruency effect follows the suggestions of Ramachandran and Hubbard (2001), as [α] and [o] are back vowels, whereas [i] is a front vowel. That is, the effect might reflect interaction in planning articulatory front/back tongue movements and manual push/pull actions, respectively. In support of this idea, the effect was also observed with consonants that require forward or backward movement of the tongue. That is, the consonant [t] in which the air-flow is blocked with the tip of the tongue was linked to push movements whereas [k] in which air-flow is blocked with the body of the tongue was linked to pull movements. Consequently, the results suggested that the front-back position of the tongue was the critical factor for the effect to arise (Vainio et al., 2015).

In general, the replicability of effects in psychology is an important topic that has gained increasing attention recently (Asendorpf et al., 2013). For example, the action-sentence compatibility mentioned above (Glenberg and Kaschak, 2002) was not replicated in a more recent study (Papesh, 2015). It is particularly important to replicate our previously found effects in another language than Finnish because there is a chance that that they are in fact language dependent. That is, the effects may not reflect language-independent interactions between processes that plan hand actions and articulatory gestures as we originally proposed. Instead, the effects might be explained by an involvement of some specific speech units in Finnish words that are semantically associated with specific hand actions. For example, the syllable [te], which was associated with facilitated push movements in the original Vainio et al. (2015) study, means “you” (plural) in Finnish. Consequently, the effect observed with this syllable might reflect a semantic association between the syllable and the hand action (e.g., pointing “at you”), in the same way as in Chieffi et al. (2009), instead of interaction between articulatory and hand movement processes.

What makes this “semantic association” explanation of our effects even more possible is the fact that words appear to be associated with related actions even in relatively abstract and metaphorical manner (e.g., Lachmair et al., 2016). As an example, Tucker and Ellis (2004) have shown that perceiving a word that represents an object that is graspable either with the precision or power grip facilitates responses performed with the grip type congruent with the word (e.g., “grape” – precision grip). Moreover, it has been shown that auditively presented meaningless syllables can evoke similar cortical activity, characterized by N400m, as typically observed with semantic processing of meaningful words (Bonte et al., 2006). Comparable mechanisms might also explain our effects. For example, the syllable [ke], which we originally assumed to be entirely meaningless, can be nevertheless a central syllable in some Finnish words that are semantically associated with the power grip (e.g., “[ke]ppi”/”stick”). In that case, one might assume that the effect reflects an implicit association between a certain word, in which a certain speech unit (e.g., [ke]) plays a central role, and the specific grip response (e.g., power grip).

However, we reasoned that if the same speech units that were used in the original studies with Finnish participants would be also associated with the same grips (i.e., precision or power) and hand movements (i.e., forward or backward) in a different language environment, our effects are not likely to be based on these kinds of semantic associations between some words and actions. It is very unlikely that these same speech units would also have a central role in some words of another language –exactly in the same way– that are in turn implicitly linked to specific grip types or forward/backward hand movements. Rather, in that case it would be more likely that the effects are based on some language-independent mechanisms that associate these meaningless speech units with specific hand actions. In that case, given the theoretical background (e.g., the mouth-hand mimicry theories; Hewes, 1973) and the empirical evidence showing the interaction between movements of articulatory organs and grasp actions (e.g., Gentilucci, 2003; Gentilucci and Campione, 2011), our original proposal that the effects reflect interaction between articulatory and hand action processes would be supported.

Consequently, in this study we addressed the generalizability of both effects for speakers of another language, namely Czech. The Czech language (a Slavic Indo-European language) was chosen as it comes from a different language family than Finnish (a Finno-Ugric Uralic language). The direct language contact has been minimal historically, and thus the language similarity and potential semantic associations are likely quite different (Benedetto et al., 2002; Kessler and Lehtonen, 2006). Also, conveniently, Czech has transparent orthography similar to Finnish; when presented with meaningless written syllables, speakers of both languages know how to pronounce the syllables consistently. Because of this we were able to use the same stimuli as in the original studies. We propose that if speech and manual processes interact at the level of articulatory gestures, both effects should be replicated. In contrast, if the effects were not replicated in Czech, it would suggest that the effects are more likely to reflect semantic associations in a specific language environment, or that the effects are not robust enough to be replicated. This would argue against our original hypothesis that the effects are based on overlapping networks between manual and articulatory gestures.

We evaluated the grip and forward/backward congruency effects in two separate experiments. In Experiment 1, we used the same paradigm as in Vainio et al. (2013) study, with utterance pairs of [kα]–[ti], [ke]–[te], and [α]–[i] to see if we can observe the grip congruency effect in another language. In Experiment 2, we used the same paradigm as in Vainio et al. (2015) study, with utterance pairs of [α]-[i], [o]–[i], and [ke]–[te] to see if we can observe the forward/backward congruency effect in another language. Besides manual responses, vocal responses were also recorded in both experiments.

## Materials and Methods

### Participants

#### Experiment 1

Nineteen native Czech speakers with no knowledge of Finnish participated in Experiment 1 (mean age 23.3 ± 3.3 years, 5 men). All participants reported normal or corrected-to-normal vision and normal hearing as well as normal hand motor functioning (3 left-handed, according to self-report). All participants gave their written informed consent for participation. This research was approved by the Ethical Committee of the Institute of Behavioural Sciences at the University of Helsinki, Finland and by the Ethical Committee of the Institute of Psychology, Czech Academy of Sciences, Czechia.

#### Experiment 2

Twenty-one native Czech speakers participated in Experiment 2 (mean age 22.0 ± 3.8 years, 4 men). All participants reported normal or corrected-to-normal vision and normal hearing, as well as normal hand motor functioning (1 left-handed, according to self-report). All participants gave their written informed consent for participation. All participants were different from the ones in Experiment 1 and reported no knowledge of Finnish language. This research was approved by the Ethical Committee of the Institute of Behavioural Sciences at the University of Helsinki, Finland and by the Ethical Committee of the Institute of Psychology, Czech Academy of Sciences, Czechia.

### Equipment, Stimuli, and Procedure

#### Experiment 1

**FIGURE 1:**
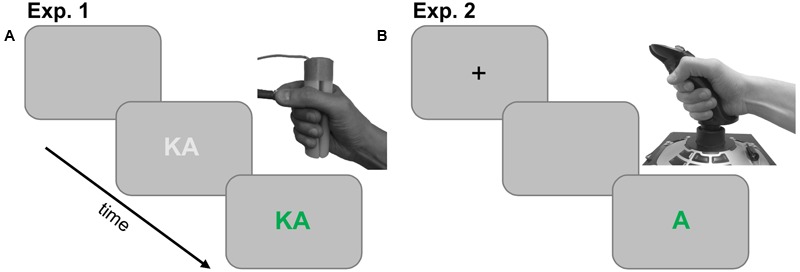
**(A)** Trial structure of Experiment 1 and illustration of the response devices. The trial started with a blank screen followed by the stimulus written in light gray. The stimulus then changed color, which was the go-signal for both vocal and manual response. **(B)** Trial structure of Experiment 2 and illustration of the joystick used for responding. The trial started with a fixation cross, followed by a blank screen and finally the stimulus written in green or blue, which the participant had to pronounce and simultaneously push or pull the joystick according to the color.

Experiment 1 was carried out at the Institute of Psychology at the Czech Academy of Sciences. The participant sat in front of a 22″ LCD-monitor, wearing a head-mounted microphone. The participant held two grip devices in their right hand. The devices were marked with blue and green tape. The cube-shaped precision grip device measured 1 cm × 1 cm × 0.7 cm and the cylinder-shaped power grip device was 12 cm in length and 3 cm in diameter. Both grip devices had an inlaid micro-switch, which gave noticeable tactile feedback when pressed. The grip signals from micro-switches were collected via computer’s parallel port. The precision grip device was held between the index finger and thumb, whereas the power grip device was held with the rest of the fingers against the palm of the hand (**Figure 1**).

The trial structure is presented in **Figure 1**. A blank gray screen was presented for 2000 ms at the beginning of the trial. This was followed by a syllable/vowel displayed in light gray color for 400 ms. After this the syllable/vowel changed color to either blue or green, which served as the go-signal for the vocal and manual responses. The color was also the response cue for whether a power or precision grip was to be executed. The stimulus remained on the screen for 2000 ms or until a response was made. Participants were instructed to respond as quickly as possible. Six participants responded with a precision grip to blue stimuli, and with a power grip to green stimuli. The color mapping was reversed for the rest of the participants. Trial presentation, grip response recording and vocal response recording were done with Presentation^®^software^1^ (Version 18.1).

It was emphasized that the presented syllable/vowel had to be uttered simultaneously while executing the manual response. Erroneous manual responses were followed by a short “beep” tone. All participants were given sufficient time to practice before the experiments so that they could properly perform the task (approximately one minute and the practice was ended when they managed to perform five or more trials in a row successfully).

The experiment consisted of three separate blocks with different utterance pairs as the stimuli. The stimulus pairs were: [kα]–[ti] written as KA and TI, [ke]–[te] written as KE and TE, and [α]–[i] written as A and I. The order of the blocks was balanced between participants. The total number of trials was 360 (30 × 2 utterances × 2 grips × 3 blocks).

#### Experiment 2

Experiment 2 was carried out at the Institute of Psychology at the Czech Academy of Sciences. The participant sat in front of a 24″ LCD-monitor, wearing a head-mounted microphone. A joystick (Logitech 3D Pro) was placed on a table in front of the participant so that it was horizontally aligned with the center of the screen. The front and back ends of the joystick were marked with blue and green tape. The joystick was operated with the right hand by moving it forward or backward (movement range from center 4.5 cm in both directions).

A blank screen was presented for 500 ms at the beginning of each trial, followed by a fixation cross for 400 ms. After that the target syllable/vowel was presented in green or blue, which acted as the go-signal. The stimulus remained on screen for 1000 ms or until a response was made. Twelve participants responded with a forward movement to blue stimuli and with a backward movement to green stimuli. The color mapping was reversed for the rest of the participants. Trial presentation and vocal response recording were done with PsychoPy software (Peirce, 2009).

It was emphasized that the presented syllable/vowel had to be uttered simultaneously while executing the joystick response. Erroneous manual responses were followed by a short “beep” tone. All participants were given sufficient time to practice before the experiments so that they could properly perform the task.

The experiment consisted of three separate blocks, with stimulus pairings [α]–[i], [o]–[i], and [ke]–[te], written as A and I, O and I, and KE and TE, respectively. The order of the blocks was balanced between participants. The total number of trials was 360 (30 × 2 utterances × 2 directions × 3 blocks).

### Data and Statistical Analysis

Onsets of the vocalizations were located individually for each trial using Praat^2^ (v. 5.3.49). Manual reaction times were measured in Experiment 1 from the pressing of the micro-switch. In Experiment 2, manual reaction times were measured from the point where the joystick first exceeded 20% of the movement range in the correct direction. The joystick was sampled at 60 Hz, so the reaction time for the 20% threshold had to be estimated by linear interpolation. Trials in which the joystick was beyond 19% of the way already at the start of trial were removed, as were those trials in which the joystick was first moved more than half-way in the wrong direction. For both manual and vocal reaction times, reactions that were over or under two standard deviations faster or slower than the participant’s mean reaction time were removed.

In Experiment 1, three participants had to be excluded due to technical issues with the data and 2.2% of the total number of trials were removed as errors, with 4.4% of grip reaction times and 4.1% of vocal reaction times removed as outliers. In Experiment 2, 5.4% of trials were removed as errors, and 4.4% of manual reaction times and 3.7% of vocal reaction times were removed as outliers.

For the statistical analysis, we performed separate repeated-measures ANOVAs for the two reaction time variables, manual, and vocal, for each experiment. Each ANOVA was a 2 × 2 × 3 design, with the factors tongue position in the utterance (front and back, e.g., [te] and [ke]), grip/direction (e.g., power and precision grip) and block. None of the comparisons violated the sphericity assumption (Mauchly’s test of sphericity all *p*’s > 0.10). We also checked the distributions of the residuals for normality and decided to analyze raw response times with no additional transformations. Interactions were assessed with pairwise comparisons, with appropriate Bonferroni corrections. For manual responses, we were interested in the pairwise comparisons between the two utterances for each grip (e.g., [kα] vs. [ti] for power grip responses). For vocal responses, were interested in the pairwise comparisons between the two grips for each utterance (e.g., power and precision grip differences for [kα] utterances). Due to the skewness of the error data, the error analysis was done with the Wilcoxon signed ranks tests by comparing the two different utterances of each block for each grip/direction. All statistical analyses were performed with SPSS, version 24 (IBM Corp. Armonk, NY, USA).

## Results

### Experiment 1

**FIGURE 2:**
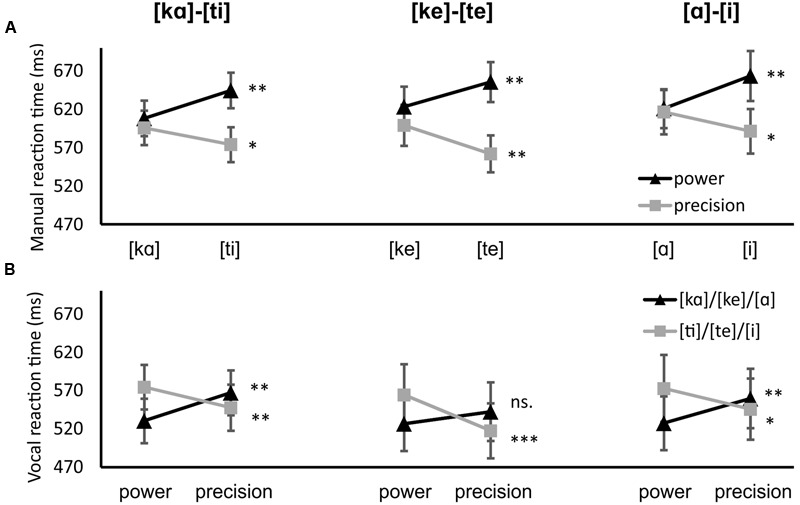
**Experiment 1 results. (A)** manual results, black lines indicate power grip responses, gray lines precision grip responses. **(B)** vocal results, black lines indicate power grip-related utterances [kα], [ke], and [α], gray lines precision grip-related utterances [ti], [te], and [i]. Error bars represent the standard error. ^∗∗∗^*p* < 0.001, ^∗∗^*p* < 0.01, ^∗^*p* < 0.05

The manual and vocal reaction time results for Experiment 1 are presented in **Figure 2** and **Table 1**. For the manual responses the interaction of tongue position and grip was significant, *F*(1,15) = 63.492, *p* < 0.001, η^2^ = 0.809. The three-way interaction between tongue position, grip and block was not significant, *F*(2,30) = 0.188, *p* = 0.830, η^2^ = 0.012, which means the interaction did not differ between blocks. The power grip responses were quicker when paired with [kα], [ke], or [α] (608, 623, and 621 ms, respectively) than when paired with [ti], [te], or [i] (645, 656, and 663 ms, *p* = 0.004, 0.002, 0.002, respectively). The precision grip responses were made faster when paired with [ti], [te], or [i] (574, 562, 591 ms, respectively) than when paired with [ka], [ke], or [a] (596, 599, 616 ms, *p* = 0.021, 0.002, 0.022, respectively). There was also a main effect of grip, *F*(1,15) = 63.492, *p* < 0.001, η^2^ = 0.809. The precision grip responses were made faster (590 ms) than power grip responses (636 ms).

**Table 1:** Mean reaction times (in ms) for each condition in Experiment 1 for both manual and vocal responses.

		[kα]–[ti]		[α]–[i]		[ke]–[te]	
Response	Grip type	[kα]	[ti]	[α]	[i]	[ke]	[te]
Manual	Precision	596	574	616	591	599	562
	Power	608	645	621	663	623	656
Vocal	Precision	567	548	560	546	542	517
	Power	530	575	527	573	527	564

Results for the vocal responses also revealed an interaction of tongue position and grip, *F*(1,15) = 27.798, *p* < 0.001, η^2^ = 0.650. [kα], [ke], and [α] were uttered more quickly when the grip was a power grip (530, 527, 527 ms, respectively) than when the grip was a precision grip (567, 542, 560 ms, *p* = 0.010, 0.158, 0.004, respectively). [ti], [te], and [i] were uttered more quickly when the grip was a precision grip (548, 517, 546 ms, respectively) than when it was a power grip (575, 564, 573 ms, *p* = 0.005, < 0.001, 0.019, respectively). The three-way interaction between tongue position, grip and block was not significant, *F*(2,30) = 0.046, *p* = 0.955, η^2^ = 0.003. There was a significant interaction between grip and block, *F*(2,30) = 6.848, *p* = 0.004, η^2^ = 0.313. In the [ke]–[te] block, utterances were generally quicker when the paired grip was a precision grip (precision 530 ms, power 545 ms, *p* = 0.036). In the other blocks, there was no difference between the grips. Lastly, there was a main effect of tongue position, *F*(1,15) = 10.511, *p* = 0.005, η^2^ = 0.412. The utterances where the tongue is positioned more backward ([α], [ke], [kα]) were uttered more quickly than the utterances where the tongue is more frontally positioned ([i], [te], [ti]) (542 vs. 554 ms, respectively).

For the error analysis, fewer errors were made when the grip was a power grip and the utterance was [kα], compared to [ti] (mean rates 1.5% vs. 3.3%, respectively, *Z* = –2.041, *p* = 0.041). Conversely, fewer errors were made when the grip was a precision grip and the utterance was [te], compared to [ke] (mean rates 0.8% vs. 2.5%, respectively, *Z* = –2.271, *p* = 0.023).

### Experiment 2

**FIGURE 3:**
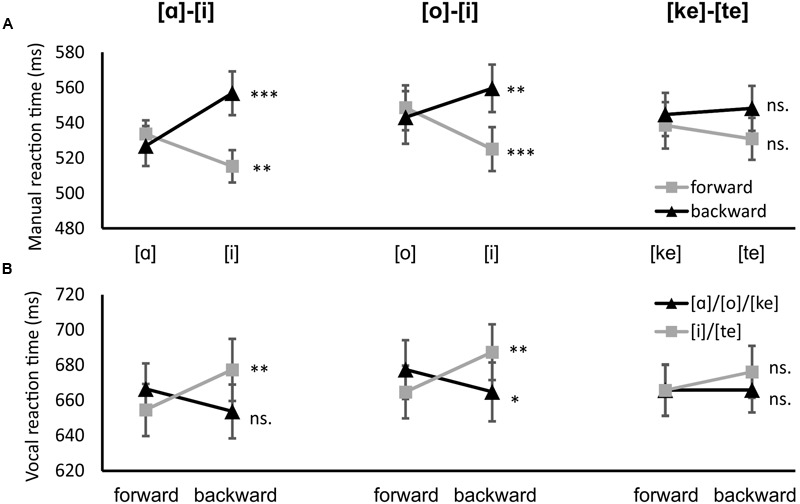
**Experiment 2 results. (A)** Manual results, black lines indicate backward responses, gray lines forward responses. **(B)** vocal results, black lines indicate backward-related utterances [α], [o], and [ke], gray lines precision grip-related utterances [i] and [te]. Error bars represent the standard error. ^∗∗∗^*p* < 0.001, ^∗∗^*p* < 0.01, ^∗^*p* < 0.05

The manual and vocal reaction time results for Experiment 2 are presented in **Figure 3** and **Table 2**. For the manual joystick responses the interaction between tongue position and direction was significant, *F*(1,20) = 16.991, *p* = 0.001, η^2^ = 0.459. However, the three-way interaction between direction, tongue position and block was also significant, *F*(2,40) = 8.102, *p* = 0.001, η^2^ = 0.288. The hand was moved faster backward when articulating [α] or [o] (527 and 543 ms, respectively) than when articulating [i] ([α]–[i] block: 557 ms, *p* < 0.001, [o]–[i] block: 560 ms, *p* = 0.009). The hand was moved faster forward when articulating [i] ([α]–[i] block: 515 ms and [o]–[i] block: 525 ms) than when articulating [α] or [o] (534 ms, *p* = 0.007, and 549 ms, *p* < 0.001, respectively). Differences in the [ke]–[te] block were not significant. There was also a significant main effect of direction, *F*(1,20) = 11.728, *p* = 0.003, η^2^ = 0.370. Forward responses were on average faster than backward responses (532 vs. 546 ms).

**Table 2:** Mean reaction times (in ms) for each condition in Experiment 2 for both manual and vocal responses.

		[α]–[i]		[o]–[i]		[ke]–[te]	
Response	Direction	[α]	[i]	[o]	[i]	[ke]	[te]
Manual	Forward	534	515	549	525	538	531
	Backward	527	557	543	560	545	548
Vocal	Forward	667	655	677	665	666	666
	Backward	654	677	665	687	666	676

Similar to the manual responses, the interaction of direction and tongue position was significant for vocal responses, *F*(1,20) = 9.514, *p* = 0.006, η^2^ = 0.322. The three-way interaction between direction, tongue position and block was also significant, *F*(2,40) = 4.592, *p* = 0.016, η^2^ = 0.187. When the hand was moved forward the vowel [i] was uttered more quickly ([α]–[i] block: 655 ms, [o]–[i] block: 665 ms) than when the hand was moved backward ([α]–[i] block: 677 ms, *p* = 0.003, [o]–[i] block: 687 ms, *p* = 0.002). The vowel [o] was uttered more quickly when the hand was moved backward (665 ms) than when it was moved forward (677 ms, *p* = 0.018). Differences when uttering the vowel [α] were not significant (backward: 654 ms, forward: 667 ms, *p* = 0.118) Differences in the [ke]–[te] block were not significant. There was also a significant main effect of tongue position, *F*(1,20) = 4.407, *p* = 0.049, η^2^ = 0.181. The backward-associated articulations [α], [o], and [ke] were pronounced more quickly on average than the forward-associated [i] and [te].

For the error analysis, fewer errors were made when the required articulation was [α] or [o] (3.8% and 4.4%, respectively) than when it was [i] ([α]–[i] block: 8.6%, *Z* = –2.534, *p* = 0.011 and [o]–[i] block: 9.0%, *Z* = –2.067, *p* = 0.039).

## Discussion

Experiment 1 replicated the grip congruency effect (Vainio et al., 2013, 2014; Tiainen et al., 2017) with Czech speakers in all three blocks, in both manual and vocal responses. Articulations of [kα], [ke] and [α] were associated with power grip, and articulations of [ti], [te], and [i] with precision grip. These results were reflected also in the error results, with fewer errors when the grip and syllable were compatible. In Experiment 2, the forward/backward congruency effect (Vainio et al., 2015) was replicated for the vowel pairs with Czech speakers. [i] was associated with faster forward movement of the hand and [α] and [o] were associated with faster backward movement. These effects were also observed in vocal responses. These results were reflected in the error rates as well, with fewer errors when the vowel was compatible with the movement direction. However, in the [ke]–[te] block the congruency was not significant, contrary to findings previously observed with Finnish participants (Vainio et al., 2015).

The current results mostly support the hypothesis that the congruency effects between grips and articulations and between horizontal hand movements and articulations are not specific to Finnish speakers. This supports the view that the effects are not caused by the sematic associations (syllables/speech units evoking certain Finnish words associated with particular hand actions). The results are consistent with the claim that the effects are based on language-independent mechanisms. In the light of previous findings concerning interactions between mouth and grasp actions (e.g., Gentilucci, 2003), we favor the view that the effect reflects interactions between processes that plan articulatory gestures and grip actions. As such, the current results support the view that the underlying mechanisms behind the effects are based on overlapping networks for hand and mouth motor functioning (Vainio et al., 2014). This might be also taken as a cautious support to gestural theories of language evolution (e.g., Rizzolatti and Arbib, 1998), or at least as support for the view that motor processes related to planning certain manual primitives might have a modulating influence on development of certain articulatory gestures (Vainio et al., 2013).

The fact that the consonant block [ke]–[te] in Experiment 2 did not replicate the forward/backward congruency effect suggests that the effect originally found with Finnish participants is likely based on semantic processes related to these syllables. This is because “te” is an outward pointing word in Finnish (plural of “you”), whereas in Czech “te” is meaningless syllable. In fact, it is likely that the interaction effect between [te] and forward hand movement reported in our original paper is based on the same mechanisms, semantically associating a specific word with a specific hand action, as the effect reported by Chieffi et al. (2009), who found similar backward and forward hand movement associations with words “here” and “there”, respectively. It is noteworthy that the effect observed by Chieffi et al. (2009) could have also been explained by articulatory properties of the words that they used in their study, because [l] in “LA” is produced by forward movement of the tongue whereas [k] in “QUA” is produced by backward movement of the tongue. As the forward/backward congruency effect was not replicated with consonants in the current study, the LA-QUA effect may indeed reflect processing of the semantic content of the word more strongly than the articulatory gestures associated with these words.

Why can the forward/backward congruency effect be observed when the task requires producing articulatory gestures for pronouncing vowels while it is absent when the task requires producing articulatory gestures for pronouncing consonants? We propose that the forward/backward congruency effect is related to an interplay between processes responsible for planning general and approximate movement direction of the hand and tongue. Production of the consonants [t] and [k] requires much more than just planning optimal movement direction for a tongue and jaw. The planning processes related to these consonants, as well as many other consonants, have to implicitly and rapidly estimate, for example, which part of the tongue (i.e., tip or back) is moved onto contact with the precise area of the velum or alveolar ridge. These planning processes also have to consider how wide area of, for example, the tongue tip is used for the articulation and how vigorously (i.e., how much strength is used) the tongue is moved onto contact with, for example, the alveolar ridge in order to make the articulation sound like [t] rather than, for example, [l]. As such, the processing demands for producing these consonants are very similar with the processing demands for producing different grips for prehension movements. Analogously for precise planning requirements related to producing these consonants, when the motor system is planning prehension actions in order to grasp different shaped and sized objects, it has to estimate, for example, the grip strength, which fingers (and how many) have to be used, and whether the finger/s has/have to be moved onto contact with the thumb or palmar surface. Given this analogy between planning demands between consonant articulation and hand shaping for a grasp, and given that the grip congruency effect was observed with the consonants [t] and [k], we propose that planning grip actions and articulation for producing consonants (at least the consonants [t] and [k]) are processed within overlapping motor networks.

Ramachandran and Hubbard (2001) theorized that words that semantically refer to pointing outward, such as “you”, are more likely to include articulatory gestures with pouting of the lips forward (i.e., rounded vowels) while words that semantically refer to pointing inward, such as “me”, are more likely to include unrounded vowels. In addition, their account assumes that articulatory organs might mimic hand gestures in words that refer to smallness (e.g., “petite” and “teeny”) by producing a “pincer gesture” with the articulatory organs using closed oral cavity and the tongue tip. There is a long research tradition in exploring interaction between speech and hand gestures (Kita and Özyürek, 2003). In general, several gesture types have been recognized: iconic gestures, deictic gestures, emblems, beat gestures and pantomimes (McNeill, 1992). In the light of this division of different gesture types, it appears that the views of Ramachandran and Hubbard (2001) are based on the assumption that, regarding the size-related gestures, certain articulatory gestures are mimicking manual gestures (e.g., the pincer grip) at the level of planning gestural iconicity. In contrast, regarding lip protrusion gestures, certain articulatory gestures are mimicking manual gestures (e.g., the manual pointing) at the level of planning deictic gestural elements. Researchers do not have a clear consensus about whether manual gestures reflect language processes that take place prelinguistically in the spatial imagery (Krauss et al., 2000) or whether manual gestures are generated by the processes that also generate speech (de Ruiter, 1998). The present study suggests that the grip and forward/backward congruency effects, that one can observe in at least two independent language systems (namely Finnish and Czech), reflect shared processes between gestural planning of articulation and hand actions.

Moreover, similarly to the findings reported by Vainio et al. (2015), in the present study both vowels [α] and [o] were associated with backward hand movement. This is somewhat in contrast with the suggestions of Ramachandran and Hubbard (2001), who theorized that words pointing outward are more likely to include articulatory gestures with pouting of the lips forward. However, we have now shown in two languages that an articulation where lips are pouted forward when producing a back vowel, namely [o], is actually associated with backward hand movement. What then seems to be the defining factor in the phenomenon is not so much the lips, but the front-back position of the tongue.

The current study shows evidence that the specific connections between hand actions and articulations observed in earlier studies are not language specific, but generalize to another language environment. Open back vowels [α] and [o] were more associated with the power grip and backward hand movement. In contrast, closed front vowel [i] was more associated with the precision grip and forward hand movement. Additionally, consonant articulations were only associated with grip actions so that the alveolar stop consonant [t] was associated with the precision grip and the velar stop consonant [k] with the power grip. These results suggest that the effects are not due to learned semantic associations but might be based on overlapping networks for hand and mouth motor functioning.

## Author Contributions

Conceived and designed the experiments: MT, JL, KT, LV, MV. Performed the experiments: JL. Analyzed the data: MT, JL. Wrote the paper and interpreted the data: MT, JL, LV, KT, MV, JS, FF. All authors read and accepted the final manuscript and agree to be accountable for the content of the work.

## Conflict of Interest Statement

The authors declare that the research was conducted in the absence of any commercial or financial relationships that could be construed as a potential conflict of interest.

## Footnotes

1 http://www.neurobs.com

2 https://www.praat.org
